# Microbial community functioning during plant litter decomposition

**DOI:** 10.1038/s41598-022-11485-1

**Published:** 2022-05-06

**Authors:** Simon A. Schroeter, Damien Eveillard, Samuel Chaffron, Johanna Zoppi, Bernd Kampe, Patrick Lohmann, Nico Jehmlich, Martin von Bergen, Carlos Sanchez-Arcos, Georg Pohnert, Martin Taubert, Kirsten Küsel, Gerd Gleixner

**Affiliations:** 1 Max Planck Institute for Biogeochemistry, Jena, Germany; 2 Nantes Université, CNRS UMR 6004, LS2N, F-44000 Nantes, France; 3 Nantes Université, INSERM U1235, TENS, Nantes, France; 4 Jena University Language & Information Engineering (JULIE) Lab, Friedrich Schiller University Jena, Jena, Germany; 5 Department of Molecular Systems Biology, Helmholtz Centre for Environmental Research – UFZ GmbH, Leipzig, Germany; 6 Institute of Inorganic and Analytical Chemistry, Friedrich Schiller University Jena, Jena, Germany; 7 Cologne Biocenter, University of Cologne, Cologne, Germany; 8 Institute of Biodiversity, Friedrich Schiller University Jena, Jena, Germany; 9 German Centre for Integrative Biodiversity Research (iDiv) Halle-Jena-Leipzig, Leipzig, Germany

**Keywords:** Carbon cycle, Microbial ecology, Mass spectrometry

## Abstract

Microbial life in soil is fueled by dissolved organic matter (DOM) that leaches from the litter layer. It is well known that decomposer communities adapt to the available litter source, but it remains unclear if they functionally compete or synergistically address different litter types. Therefore, we decomposed beech, oak, pine and grass litter from two geologically distinct sites in a lab-scale decomposition experiment. We performed a correlative network analysis on the results of direct infusion HR-MS DOM analysis and cross-validated functional predictions from 16S rRNA gene amplicon sequencing and with DOM and metaproteomic analyses. Here we show that many functions are redundantly distributed within decomposer communities and that their relative expression is rapidly optimized to address litter-specific properties. However, community changes are likely forced by antagonistic mechanisms as we identified several natural antibiotics in DOM. As a consequence, the decomposer community is specializing towards the litter source and the state of decomposition (community divergence) but showing similar litter metabolomes (metabolome convergence). Our multi-omics-based results highlight that DOM not only fuels microbial life, but it additionally holds meta-metabolomic information on the functioning of ecosystems.

## Introduction

The importance of dissolved organic matter (DOM) leaching from the litter layer for terrestrial carbon cycling is unequivocal^1–4^. However, the role of DOM goes beyond its function as a readily-available substrate for decomposer communities, and its role as a microbial meta-metabolome that contains signatures of ecosystem functioning is less explored^5^. The latter function has been suggested following rapid structural adaptations of decomposers during early-stage litter decomposition, when the substrate composition changes fast as well^6^. On the local scale, this leads to litter-specific decomposer community profiles^7–9^. On the landscape scale, however, environmental factors like pH^10,11^ and soil geochemistry^12^ at the litterfall location are suggested as major drivers of microbial community structure.

How changes in substrate composition and environmental factors enforce functional adaptation of microbial communities has yet to be determined. Recently, it has been suggested that competition could be a major facilitator of both structural change and functional adaptation within topsoil microbial communities on a global scale^13^. Mechanisms of attack and defense, that happen on a molecular level between various actors across kingdoms, are being unraveled at unprecedented levels of detail^14,15^. Recent studies suggest a vast potential of soil microorganisms for the biosynthesis of antibiotics and targeted toxins^15,16^. As these substances are secreted, DOM could be an ideal medium to trace active competition functions on a community level and their relationship to changing substrates and environments^4,17–19^.

In this study, we report on a litter decomposition experiment focusing on rapid functional adaptations of microbial decomposer communities to their substrate within the first weeks of litter decomposition^20^. The potential adaptation is assessed via combined analyses of litter leachates (DOM HR-MS, metabolome LC–MS) and community profiles (16S rRNA, metaproteome). We hypothesize that at the early stage of litter decomposition, the native decomposer communities (a) are already well adapted to the litter’s properties and (b) rapidly further optimize their functions to address changes in substrate composition as decomposition progresses. Furthermore, we hypothesize that the progression and mode (synergistic or antagonistic) of community-level functional adaptation are well recorded in the molecular composition of DOM. We test our hypotheses on three major vegetation types: broadleaf forest, evergreen forest and grassland. We account for site-specific factors that could affect the leaf metabolome and decomposer communities by sampling the same vegetation on two sites with distinctly different soil properties and pH^21^. Our laboratory setup excludes the possibility for hostile colonization of the litter by soil microorganisms, allowing us to attribute observed changes in community composition and functioning to adaptations within decomposer communities that natively live on the litter.

## Methods

### Sampling sites

Senescent beech (*Fagus sylvatica*) and oak leaves (*Fraxinus excelsior*), pine needles (*Pinus sylvestris*) and grassland litter were sampled on the AquaDiva research site in the Hainich national park (Thuringia, Germany) and the Linde research station of the Zwillenberg Tietz foundation near Märkisch Luch (Brandenburg, Germany) (Supplementary Fig. S1). The plant litter sampling in Hainich was carried out with permission of the Hainich national park administration and in Linde with permission of the Zwillenberg Tietz foundation as the landowner. The Hainich site features near-neutral soil water (pH 6), whereas in the Linde site the soil water is more acidic (pH 4.9). The differences in pH arise due to carbonate buffering in the Hainich soil, which developed on a bedrock of marine carbonate sediments from the Triassic era^22^. In contrast, the Linde soil developed on siliciclastic material from the Weichselian Glaciation during the Pleistocene. Thus, our site replication covers a wide range of the natural pH-related variability in soil microbial community composition^12^, which has been suggested to affect the secondary metabolite profile of the plant.

### Sampling procedure

**Figure 1:**
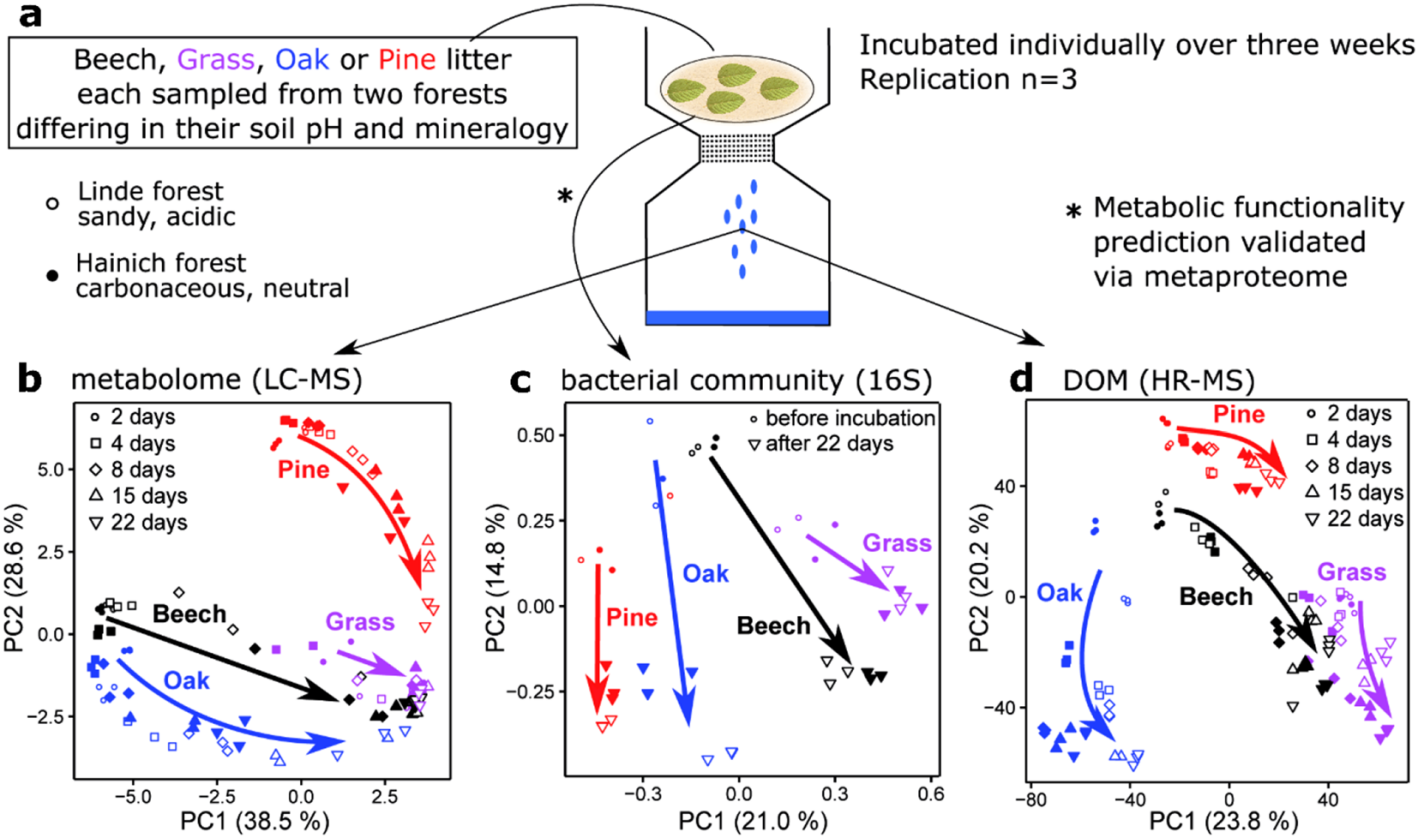
(**a**) Conceptual overview of the study design. (**b**) PCA of the LC–MS metabolome results indicates chemical convergence. (**c**) PCA of the 16S rRNA gene barcoding data indicates divergence and litter-specific community evolution. (**d**) PCA of the HR-MS data reveals a divergence between the tree-derived DOM, yet convergence between the beech and grass-derived DOM after ~ 8 days.

The plant litter was collected in October 2017 at both sites as soon as the start of litterfall was observed. Tree litter was collected from the forest floor at single-species stands in respective radii of ~ 50 m to account for very local variability in both forests. Grassland sites held a diverse community of grass and herb species, but only the dominant grass species were sampled. The sampling of both sites was completed within two consecutive days to achieve similarly minimal exposure of the litter to soil microbial communities. Care was taken to pick up only recently fallen litter from the top of the litter layer. After sampling, the material was transported to the lab and immediately dried at 40 °C for 72 h. As we cannot exclude that transport and drying might have reduced the potential for site-specific processes to occur, we checked the 16S taxonomic profiles of the litter both wet, directly from the forest, and after drying. They are summarized as dots Fig. 1c and their close proximity indicates that transport and drying were only minor contributors to the overall variability.

### Experimental setup

The dried plant litter was coarsely cut and separately mixed with pre-combusted (500 °C/5 h) acid-washed sand at a ratio of 3 g plant material per 100 g final mixture to allow proper through flow of water. The sand and plant material mixtures were placed inside 250 mL filter holders (Nalgene, Thermo Fisher Scientific, Waltham, MA, USA) above a GF/D (2.7 µm) and a GF/F (0.7 µm) glass fiber filter. Both filters were pre-combusted (500 °C/5 h) and the plastic filter holders were pre-autoclaved (121 °C/20 min). Sand and litter mixtures were set up in triplicate, resulting in 24 parallel decomposition setups (four plant litter types from two sampling sites replicated three times). We set up three blanks containing only sand with no plant material, which were treated and sampled equally, showing no measurable TOC release or pH effect. The sand and litter mixtures were wetted with ultrapure water and checked daily for continuous wetness. After 2, 4, 8, 15 and 22 days, 100 mL of ultrapure water was added to leach DOM from the litter. During this time, prolonged periods of stagnant water were avoided by accelerating through flow using a soft vacuum of ~500 mbar for a maximum of five minutes. Immediately after the last leachate sampling, the sand and litter mixtures were sampled destructively into sterile falcon tubes for microbial community analysis.

### DOM extraction and direct-injection HR-MS analysis

DOM was extracted from the leachate samples using a common solid-phase extraction protocol over 1 g PPL resin^23^. For direct-injection HR-MS analysis, the concentration of the extracts was adjusted to exactly 20 mg C/L in a 1:1 water and methanol solvent mixture. 100 µL of DOM extract were directly injected into a continuous flow of 20 µL/min water and methanol (1:1) using an autosampler (Thermo Fisher Scientific). Measurements were carried out on an Orbitrap Elite mass spectrometer (Thermo Fisher Scientific) with a mass resolution of 240,000 m/m. Electrospray ionization (ESI) was run in negative ionization mode with and ESI needle voltage of 2.65 kV. 100 scans of m/z 175–1000 were acquired per sample with detailed settings and sum formula assignment as previously described in^19,24^. Only masses with a signal/noise ratio (S/N) > 4 were analyzed further. Masses that were detected in blanks were not considered for further analysis.

Metabolic pathway information was gathered from KEGG using their application programming interface at https://www.kegg.jp/kegg/rest/ (access date: 2019-11-04)^25^. 20% of the assigned sum formulae had a match in the KEGG database. On average, there were 2.9 structure suggestions per matched sum formula. We summed the relative intensities of the sum formulas that matched with KEGG, and grouped them by their respective metabolic pathways. As some sum formulas had multiple matches, only one intensity contribution per sum formula was allowed in each pathway group. We note that the annotation of sum formulas in KEGG is prone to contain some amount of individual false positive matches due to the inability to differentiate between isomers. However, the likelihood of an incorrect pathway assignment decreases as more different sum formulas are assigned to the same pathway. In addition, we observed that sum formulas, which had many matches in KEGG, oftentimes were assigned multiple times to the same pathway as some isomeric structures have similar biochemical properties. This further reduces the likelihood of incorrect pathway assignments being reported.

### DOM LC–MS analysis

Metabolic profiles were obtained by injecting 1 µL of each leachate extract (section above) on a LC-(Ultimate 3000) Q-Exactive (MS) system (Thermo Fisher Scientific). Separations were achieved on a Accucore C18 column (100 × 2.1 mm, 2.6 µm, Thermo Fisher Scientific) by using a mobile phase of A: Water (with 0.1% formic acid (FA) and 0.2% acetonitrile), and B: Acetonitrile (with 0.1% FA) as follows: 0–0.2 min 100% (v/v) A isocratic, 0.2–8 min a gradient to 100% (v/v) B, 8–11 min 100% (v/v) B isocratic, 11.0–12.0 min a gradient to 100% (v/v) A, 12.1–14 min 100% (v/v) A isocratic. The flow rate was set to 400 µL/min. Electrospray ionization was set to negative ionization mode (spray voltage of 2.5 kV). Full MS acquisition with a resolution of 70000 m/m, automatic gain control (AGC target) of 3 × 10^6^, and a scan range of 100–1500 m/z. Raw data files were then converted to mzXML format by using MSConvert (ProteoWizard 3.0). Data were processed with XCMS and CAMERA packages under R 3.3.3 environment. Afterward, the peak matrix was filtered by removing background ions from negative control samples and features with a relative standard deviation (RSD) of > 20%.

### Extraction of DNA

DNA was extracted from the initial plant litter samples (wet from the forest and after drying) as well as from the sand-litter-mix after 22 days of incubation. For each sample, 0.5 g of plant litter or sand-litter-mix were weighed into a silica beads tube, and 375 µL of 120 mM phosphate buffer (pH 8.0), 125 µL of TNC buffer (0.5 M Tris–HCl, 0.1 M NaCl, 10% CTAB (hexadecyltrimethylammonium bromide) pH 8.0), and 500 µL of PCI (phenol:chloroform:isoamylalcohol 25:24:1, pH 8.0; Roth, Karlsruhe, Germany) were added. Bead beating was performed for 30 s at 6.5 m/s in an MP Biomedicals FastPrep-24 (Fisher Scientific, Schwerte, Germany), followed by centrifugation at 13,000×*g* for 5 min. The supernatant was transferred to a new tube, and the extraction from the plant litter or sand-litter-mix was repeated twice as described. The pooled aqueous phases were extracted once more with PCI and once with chloroform:isoamylalcohol 24:1 (Roth), followed by an addition of 1 µL 20 µg/µL glycogen and 1.5 mL polyethylene glycol solution (30% PEG 8000, 1.6 M NaCl) for DNA precipitation. Following 2 h of incubation at room temperature, samples were centrifuged at 14,000×*g* for 90 min at 4 °C, DNA pellets were washed with 75% ethanol and centrifuged as above for 20 min, and resuspended in 100 µL TE buffer (10 mM Tris–HCl, 1 mM EDTA, pH 8.0).

### Amplicon sequencing of bacterial 16S rRNA genes

A polymerase chain reaction targeting the V3 to V5 region of bacterial 16S rRNA genes was performed using primer pair Bact_341F/Bact_805R and HotStarTaq Mastermix (Qiagen, Hilden, Germany) as previously described^26,27^. Purification of amplicons was done using NucleoSpin Gel & PCR Clean-Up Kit (Macherey–Nagel, Düren, Germany). Libraries for amplicon sequencing were prepared using the NEBNext Ultra DNA Library Prep Kit for Illumina (New England Biolabs, Frankfurt, Germany) following the manufacturer’s instructions. They were purified using AMPure XP beads (Beckman Coulter, Krefeld, Germany). Amplicon sequencing was carried out using a MiSeq Illumina platform (Illumina, Eindhoven, The Netherlands) with v3 chemistry. Analysis of raw sequence data was performed in mothur (v. 1.39) following the mothur standard operating procedures as previously described^28–30^. Sequences were binned to OTUs with a 3% identity cutoff, and OTUs were classified using the SILVA reference database release SSU 132^31^.

### Metaproteomics

Protein extraction and sample preparation for LC–MS/MS measurements were performed according to previously published protocols^32^. In brief, the proteins were prepared by SDS–polyacrylamide gel electrophoresis (SDS-PAGE) for sample decontamination and in-gel digested with 0.5 µg trypsin (Sigma-Aldrich, St. Louis, USA), overnight. The extracted peptides were desalted using ZipTip filter (Thermo Fisher Scientific) following the manufacturer’s instructions and analyzed using liquid chromatography (HPLC, Ultimate 3000 RSLCnano, Dionex/Thermo Fisher Scientific, Idstein, Germany) coupled via a TriVersa NanoMate (Advion, Ltd., Harlow, UK) source in LC chip coupling mode with a Q Exactive HF mass spectrometer (Thermo Fisher Scientific). The samples were measured according to the settings outlined in Starke et al.^33^. The acquired raw data were searched by Sequest HT in Proteome Discoverer v2.1 (Thermo Fisher Scientific) against an *in-silico* protein database containing all bacteria (> 10^6^ sequences), downloaded 2017 from Uniprot. We considered only proteins with a false-discovery rate of < 1%.

### Data analysis

Principal components analysis and visualizations were performed in R3.6^34^ using packages vegan^35^, and ggplot2^36^. Weighted correlation network analysis (WGCNA) was performed on the HR-MS DOM data using an interactive web application available at https://shiny-bird.univ-nantes.fr/app/Mibiomics^37^. In the WGCNA, we used Pearson correlation to build the similarity matrix between all combinations of sum formulas in our data set. A signed adjacency matrix (*A*) was created as ${A}_{i,j}={\left(0.5*\left(1 +cor\right)\right)}^{p}$ with *cor* as the Pearson correlation value and *p* = 9 to achieve a scale-free topology. Negative correlations result in low adjacencies. Hierarchical clustering of the node dissimilarities identified subnetworks, called *modules* in WGCNA. The WGCNA dissimilarity matrix was scaled into a three-dimensional representation via classical multidimensional scaling and visualized with R package rgl^38^.

## Results

### Litter and decomposer community evolution

A principal component analysis (PCA) of our litter metabolome (LC–MS) revealed major initial differences between the broadleaf and coniferous litter as well as the grassland litter (Fig. 1a,b). Over the 22 days of decomposition, the metabolome composition of all three tree litter types converged towards the grassland litter, which showed little temporal evolution after 8 days. The PCA did not reveal significant differences between the two sampling sites. Our data show that (i) the metabolome is initially litter-specific, rather than site-specific, (ii) the tree litter metabolome changes stronger than the grassland litter over the 22 days of incubation and (iii) metabolome converges rapidly, reaching high similarity already after 22 days. The rapid convergence suggests that the initial differences between the litter metabolomes are rapidly being addressed and removed by the respective decomposer communities.

Amplicon sequencing of bacterial 16S rRNA genes on DNA showed distinct differences between the community structures on the litter types before and after the 22-day incubation (Fig. 1c). This indicated high litter-specificity throughout the decomposition process. Again, there was only minor variability related to the sampling sites. In contrast to the convergence of the litter metabolome profiles (Fig. 1b), the compositions of the bacterial communities diverged (Fig. 1c). A pairwise Euclidean distance matrix of the 16S data showed that during over 22 days of decomposition, the dissimilarity between the communities on the four litter types increased by ~ 6% (Supplementary Table S1). Our data show a divergent development of litter-specific bacterial communities during early-stage decomposition, suggesting increasing substrate-dependent specialization.

Direct infusion HR-MS analysis of DOM revealed a high initial similarity between the samples of oak, pine and beech litter (Fig. 1d). From there, the composition of the tree-derived DOM diverged until day 8. The grassland DOM did not share the initial similarity with the tree-derived DOM, but it converged with the beech signal after ~ 8 days of decomposition. From day 8 to 22 we observed that the paths of temporal change of all litter types were close to parallel in the PCA, suggesting they might undergo similar transformation processes.

The metabolome and 16S data were dominantly converging and diverging, respectively, but the HR-MS DOM data contained both converging and diverging trends. This suggests that DOM contains information on both the litter metabolome and the decomposer communities. This advocates for a more detailed visualization with the aim of assigning the individual molecular components of DOM to their most likely sources.

### Weighted molecular network of DOM

**Figure 2:**
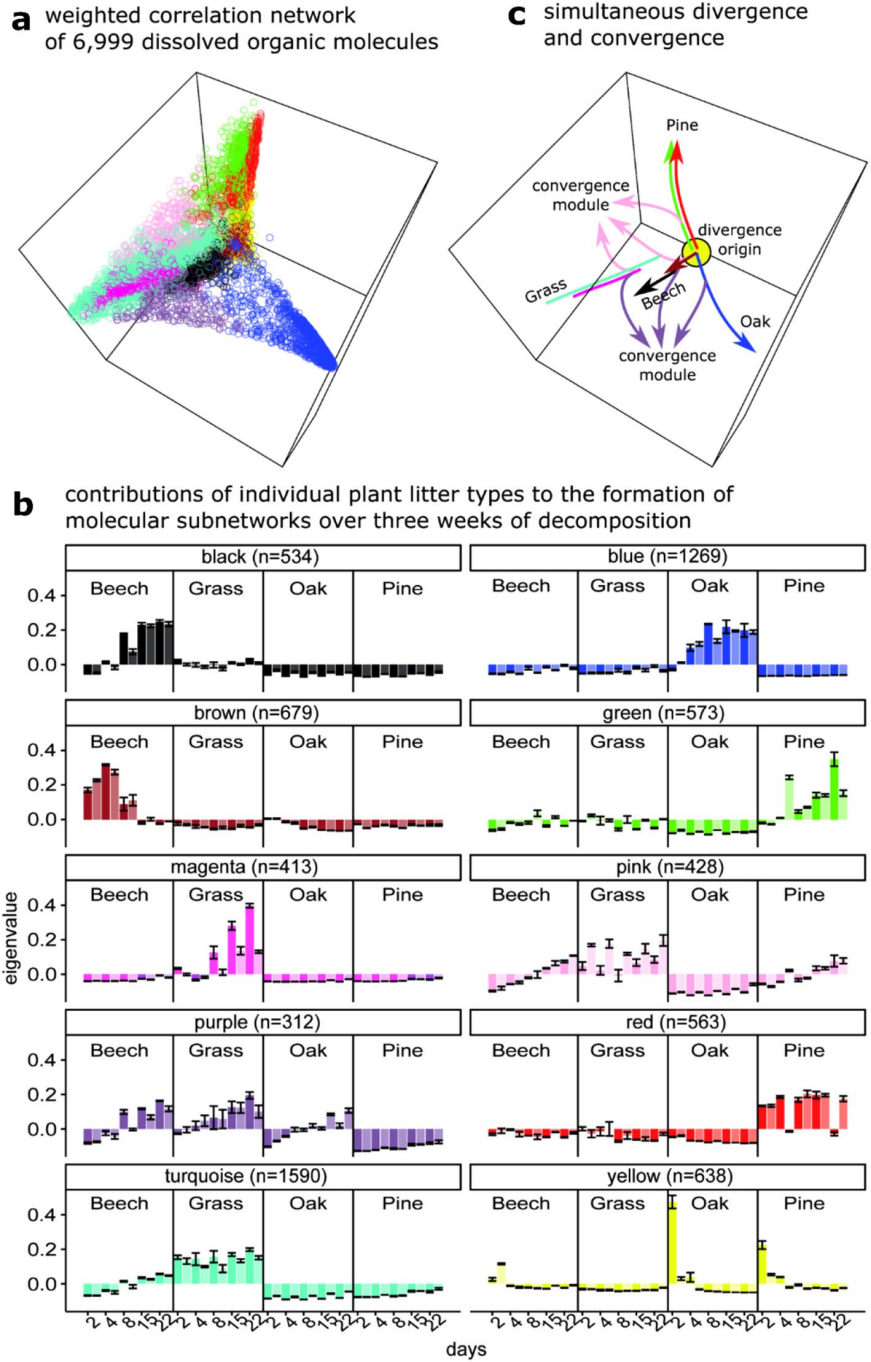
(**a**) Weighted correlation network analysis (WGCNA) of a combination of 6999 molecular entities in DOM scaled into three dimensions. (**b**) Contributions of the DOM samples to the network modules. The samples are grouped by litter type. Each bar summarizes three biological replicates. A lighter shade identifies bars representing samples from the Linde forest; a darker shade samples from the Hainich forest. (**c**) Summary graph indicating simultaneous divergence and convergence during litter decomposition.

A weighted correlation network analysis (WGCNA) of our HR-MS DOM data produced a weighted graph composed of 6999 nodes (Fig. 2a). Therein, each node represents a distinct molecular component, which is described by a sum formula and its relative abundance in the 120 HR-MS spectra. The topological position of two nodes within the network reflects the similarity of the individual molecules’ relative abundances, meaning: the closer two nodes plot together, the higher the similarity between the relative abundance patterns of the underlying molecules during the litter decomposition experiment.

The three-dimensional shape of the total network is tetrahedron-like, suggesting 4 potential end member components within the DOM composition. These 4 compositional end members could potentially correspond to the 4 litter types, but also to the 4 temporal stages after the initial sampling. To identify whether the input litter types and the temporal evolution shaped the network structure, we analyzed the contribution of each sample to subsections of the network, so-called network modules (Fig. 2b). In the WGCNA, the module eigenvalues depict these contributions.

The yellow network module represented the initial conditions for the tree species beech, oak and pine. This suggests that at the initial stage of decomposition, the DOM leaching from the 3 tree species was highly similar, but was distinct from the grassland DOM. Seven of the ten network modules had major contributions from one litter type only (in Fig. 2b beech: black and brown, oak: blue, grass: magenta and turquoise, pine: green and red). In many of these modules, the eigenvalues of their respective litter types were increasing along the time series. We conclude that that following an initial similarity, litter-specific molecular patterns were forming and consolidating within the composition of DOM.

In addition, the WGCNA identified two modules (pink and purple) that indicate partial convergence. The pink module captures mixed contributions from the beech, grass and pine litter that increase with time. The purple module shows a highly similar pattern, but for beech, grass and oak litter. Within the whole network, these two convergence modules filled the topological space between the modules that were highly litter-specific and located in the edges of the tetrahedron (Fig. 2c). The WGCNA revealed that divergence and convergence occur simultaneously in DOM during early-stage litter decomposition. This suggests that common and litter-specific decomposition functions are simultaneously being employed by the decomposer community, and that their metabolic expression is recorded by distinct sets of molecules within DOM. Furthermore, our findings open the question of why the sampling site was not an important factor in shaping the DOM composition in our experiment. We hypothesize that decomposer communities optimize their functional expression towards the litter type irrespective of their taxonomic structure, which is commonly observed to vary with pH and geochemistry between sites^12^.

### Predicting decomposer community functioning

**Figure 3:**
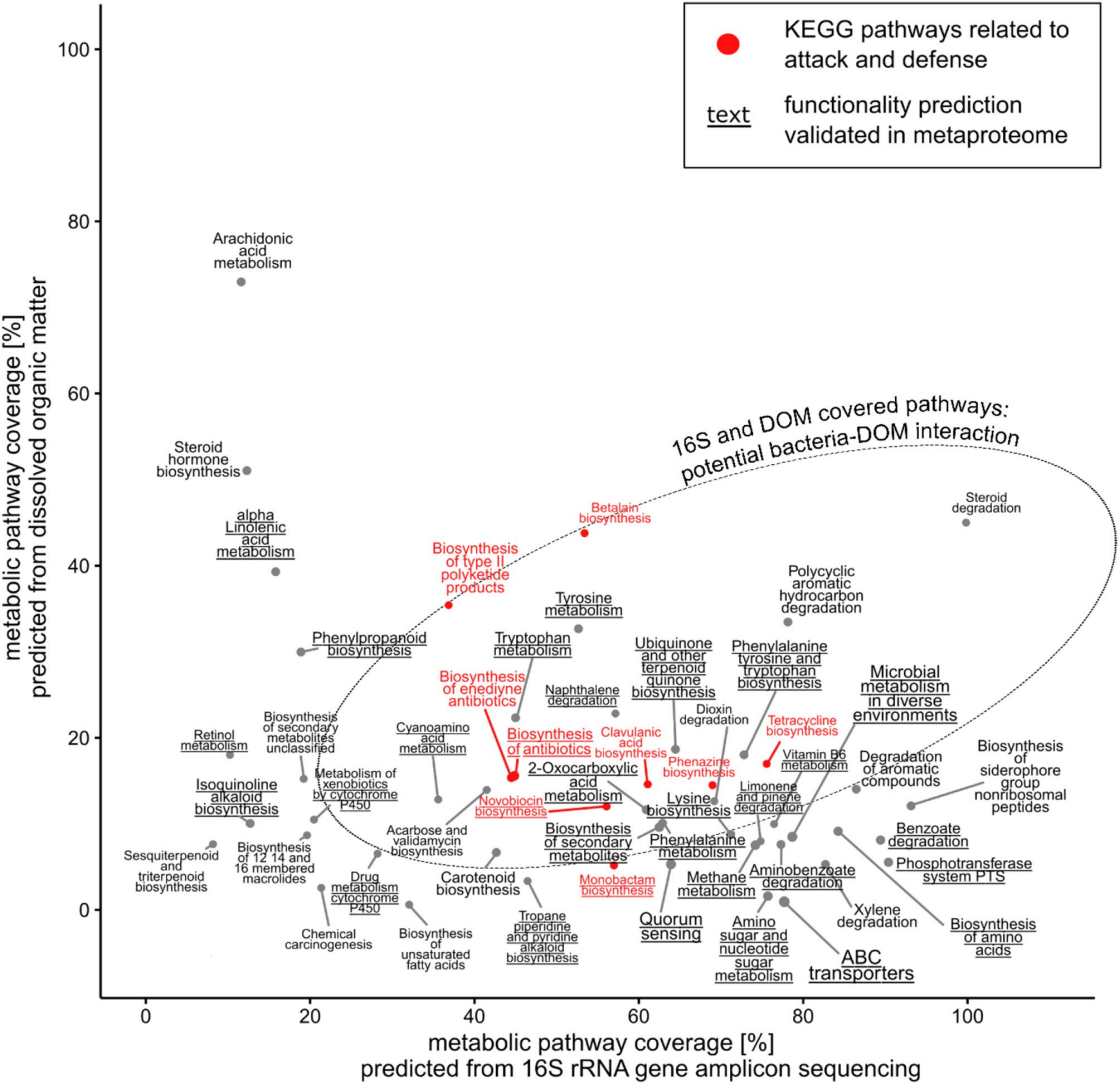
Map of decomposer community functioning based on average coverages in DOM HR-MS and 16S rRNA gene amplicon sequencing. Underlined pathway predictions were additionally validated by metaproteomics. Pathways highlighted in red indicate competitive metabolism through antibiotics release and resistance.

Functional predictions based on the 16S rRNA gene provide an approximation of the functional profiles of microbial communities^39^. Our findings above showed that DOM additionally contained information on decomposer communities and their substrates. Correspondingly, central pathways of microbial maintenance such as ‘ABC transporters’, ‘quorum sensing’ and ‘phosphotransferase system PTS’^40–42^ are highly covered in our 16S data, but have only very low molecular coverage in DOM (Fig. 3 and Supplementary Table S2). In contrast, the pathway ‘phenylpropanoid biosynthesis’, which refers to lignin precursors^43^, is highly covered only in DOM, indicating the leaching and residual presence of litter-derived molecular components.

We expect that functional interactions between decomposer communities and their litter substrates could most likely be found among pathways that have comparable coverage in both DOM and 16S. We find that several pathways, which are highly covered both in DOM and 16S, indicate mechanisms of attack and defense. Not only did we find high coverages in the overview pathway ‘biosynthesis of antibiotics’, but there were many more specific pathways as well. One of the most highly covered pathways, ‘betalain biosynthesis’ refers to a compound that has been suggested to enable defense against pathogenic fungi^44^. While our current analysis does not cover fungal actors, previous investigations suggest that antagonism between bacteria and fungi is likely to occur as a result of substrate competition^45^. The pathway ‘tetracycline biosynthesis’ refers to a class of antibiotics, that are effective against a wide range of Gram-positive and Gram-negative bacteria^46^. The pathway ‘phenazine biosynthesis’ hints at microbial defense, as phenazine production in biofilms has been shown to promote antibiotic tolerance^47^. The complexity of these molecular attack and defense mechanisms is surprisingly high, seeing as the pathway ‘clavulanic acid biosynthesis’ indicates the production of a compound, that is not an antibiotic itself, but is used to overcome β-lactam resistance in bacteria that secrete β-lactamase^48^. β-lactamase otherwise inactivates many common antibiotics. Our findings suggest that decomposer communities may employ highly sophisticated attack and defense mechanisms during the early stages of litter decomposition.

Since rapid community succession during litter decomposition has been previously shown^49,50^, we hypothesize that the structural changes might be driven by competition, which is molecularly exerted via the secretion of antibiotics into DOM. Our 16S rRNA gene data reveal that *Proteobacteria* were the dominant phyla on all four litter types (Supplementary Figs. S2 and S3). However, their relative abundance decreased by ~ 15% on average over the 22-day incubation, whereas *Actinobacteria* rose by approximately the same amount. Relative abundances of *Actinobacteria* species have previously been shown to increase during litter decomposition and have been described as secondary generalist decomposers^49,51^. This suggests that some species within the initially dominant phylum of *Proteobacteria* could be under increasing competitive pressure, giving way to *Actinobacteria* species, who are generally known for their competitive abilities such as the production of a variety of antibiotic compounds^52^.

Functional predictions based on the metaproteome supported our hypothesis of active competition between the major bacterial phyla in our experiment (Supplementary Table S3). The metaproteome revealed multiple defense functions of *Proteobacteria* and *Firmicutes*, namely beta-lactam resistance and cationic antimicrobial peptide (CAMP) resistance^53^. Monobactam and novobiocin biosynthesis could be assigned to *Proteobacteria*. Both are potent antibiotics^54,55^ and were predicted by our HR-MS and 16S rRNA gene data as well (Fig. 3). In accordance with the decrease in *Proteobacteria*, the coverage of the monobactam and novobiocin biosynthesis pathways in the HR-MS data also decreased over time during our incubation (Supplementary Table S2). This finding suggests a distinct effect of competitive metabolism and decomposer community change on the molecular composition of DOM in litter leachates.

We find that the coverage of metabolic pathways (proportion of detected molecules and functions relative to pathway size) in both HR-MS and 16S was surprisingly similar between the litter types, time points and sampling sites. Within the whole decomposition series, the standard deviations of the coverages per pathway were only about ± 6% and ± 4% on average for the DOM and 16S data, respectively (Supplementary Table S2). This indicates that the early-stage decomposition process of broadleaf, evergreen and grass litter requires many of the same functions.

## Discussion

We found that the qualitative (presence/absence-based) differences in pathway coverage between the litter types, time points and sites in our decomposition experiment were marginal. This finding supports a prevalent theory in microbial ecology, which suggests high functional redundancy in terrestrial microbial communities, especially in the context of organic matter decomposition^56,57^. In contrast, our weighted network analysis of DOM, which is based on relative abundances, suggests the emergence of highly litter-specific molecular patterns (Fig. 2). This finding supports theories of metabolic specialization during decomposition^8,58^.

**Figure 4:**
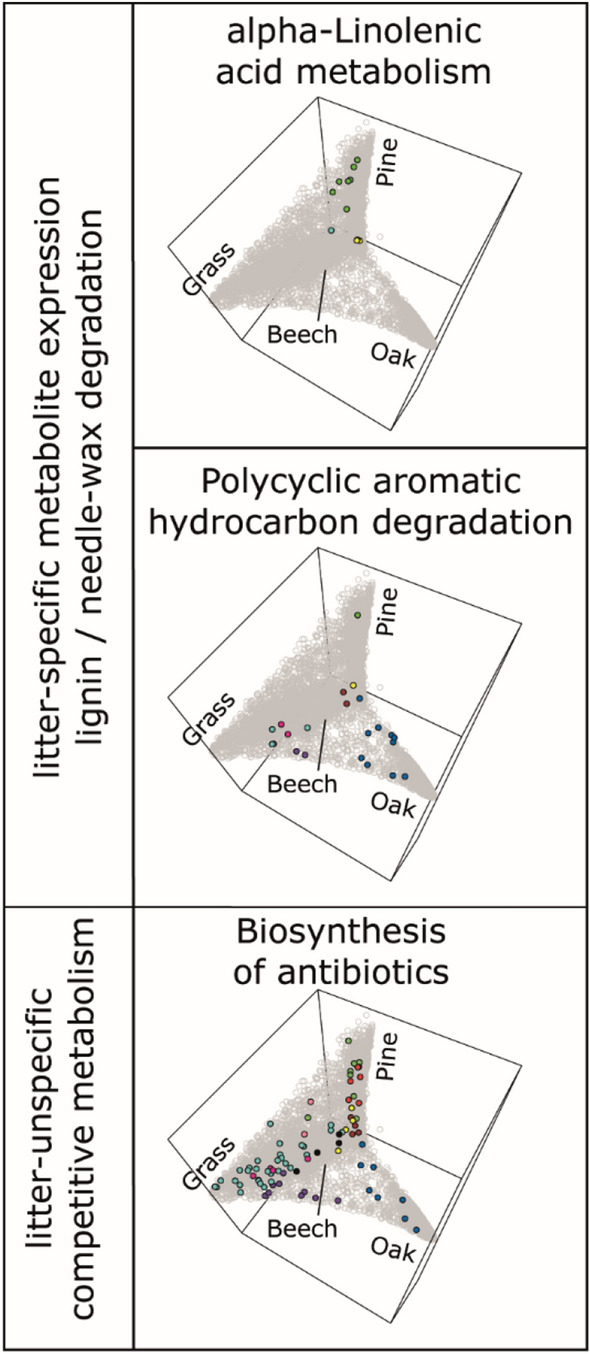
Molecular entities, that were annotated into metabolic pathways, are highlighted within the same weighted correlation network of DOM. Pathways, that are indicative of the degradation of needle waxes (top) and lignin (middle) are highly and little expressed in the pine litter, respectively, suggesting specifically optimized degradation strategies. Molecules in the pathway ‘biosynthesis of antibiotics‘ (bottom) are ubiquitously distributed.

To unify specialization and functional redundancy during litter decomposition, we integrated our molecular network and metabolic pathway prediction (Supplementary Table S4). We find that the distribution of the functional metabolites in the network differed sharply between pathways (Fig. 4). Molecules belonging to the pathway ‘alpha-linolenic acid metabolism’ are almost exclusively found in the green module, whose members have high relative abundances in the pine litter. The pathway ‘polycyclic aromatic hydrocarbon degradation’ however, shows an opposite pattern, being under-represented in the pine litter modules (green and red). Even though we have found previously that the presence/absence-based coverages of both pathways are similar between the litter types, their relative expression patterns are highly litter-specific. In this decomposition study the litter was coarsely cut and as a result the pine needles were structurally intact (Supplementary Fig. S1). Therefore, the metabolization of the needle wax covers, which have been shown to contain alpha-linolenic acid, could have required priority in the decomposition process of the pine litter^59^. The breakdown of the polymerized aromatic hydrocarbons from lignin could, as a result, be delayed during pine needle degradation. Our findings suggest that even though the metabolic potential of decomposer communities is functionally redundant from a qualitative (presence/absence) perspective, the relative expressions of decomposition functions are optimized towards the properties of the immediate substrate.

For decomposer communities it might be less important *what* has to be done, because many functions have to be employed in a similar manner on various litter types and are therefore redundantly distributed. It could be more important *how much* of the respective function has to be performed locally at each point in time and how well the respective actors within the decomposer community are able to perform these tasks.

We did not find a litter-specific association to explain the competitive aspects of the decomposition processes. The molecular entities, which were annotated into the pathway ‘biosynthesis of antibiotics’, were spread ubiquitously throughout the weighted network graph. This suggests that attack and defense could be universal mechanisms among decomposer communities to compete, adapt and optimize for their substrate, supporting proposed global patterns of microbial competition in the topsoil^13^.

## Conclusions

In this investigation, we highlight the potential of integrating heterogeneous data from multi-omics analyses to reveal functional interactions between microorganisms and dissolved organic matter (DOM). Our study expands on recent advances in understanding how microbial communities adapt to their substrate and how this shapes the process of plant litter decomposition. We identify groups of hundreds of molecules in DOM that together are highly indicative of individual plant litter types and their stage of decomposition. In the future, refining such DOM-based indicator patterns could directly inform about the metabolization and fate of plant-derived carbon in soil and contribute to assessments of the function and health of terrestrial ecosystems. Our findings suggest that bacteria secrete a variety of natural antibiotics in an effort to compete against other bacteria or fungi within the decomposer community. Competitive pressure likely drives constant adaptation and optimization of decomposer community functioning.

## Supplementary Information

Supplementary Table S1.

Supplementary Table S2.

Supplementary Table S3.

Supplementary Table S4.

Supplementary Figures.

## Data Availability

Raw Illumina MiSeq sequencing data have been deposited in the Sequence Read Archive of NCBI under Accession Numbers SRR11837071–SRR11837111. Raw metabolome data have been deposited in the MetaboLights database under accession number MTBLS1885. Raw HR-MS DOM and metaproteome data are available from Edmond, the Open Research Data Repository of the Max Planck Society, under 10.17617/3.4c.
