# Supplementary material for: Microbial community functioning during plant litter decomposition
Source: Sci Rep. 2022 May 6;12:7451. doi: 10.1038/s41598-022-11485-1 (PMC9076648; doi:10.1038/s41598-022-11485-1)
Supplement: Supplementary file 5 — Supplementary Figures. [file 41598_2022_11485_MOESM5_ESM.docx]

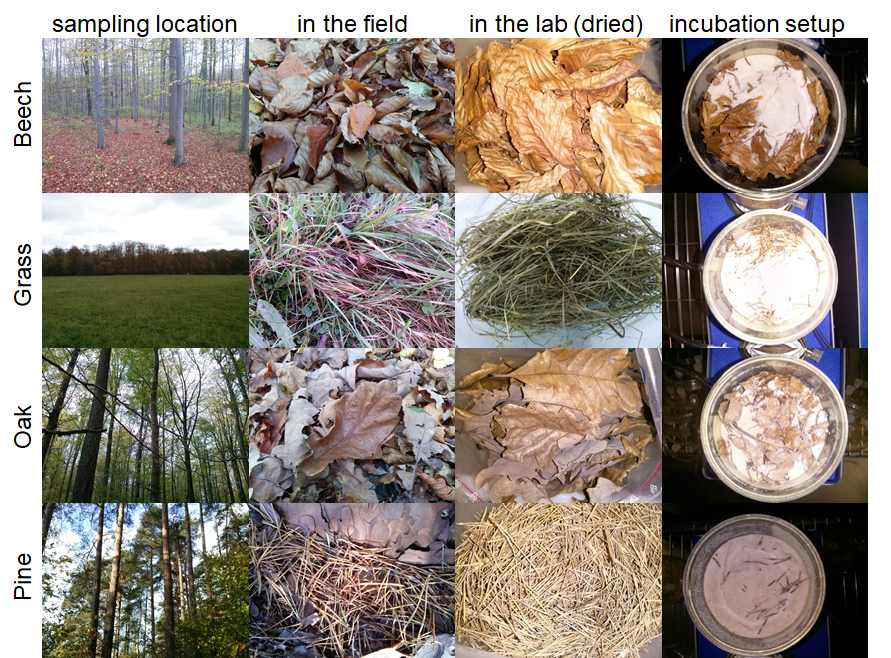


**Supplementary Figure S1** Photographic overview of litter sampling in the Hainich forest, sample preparation and incubation setup.


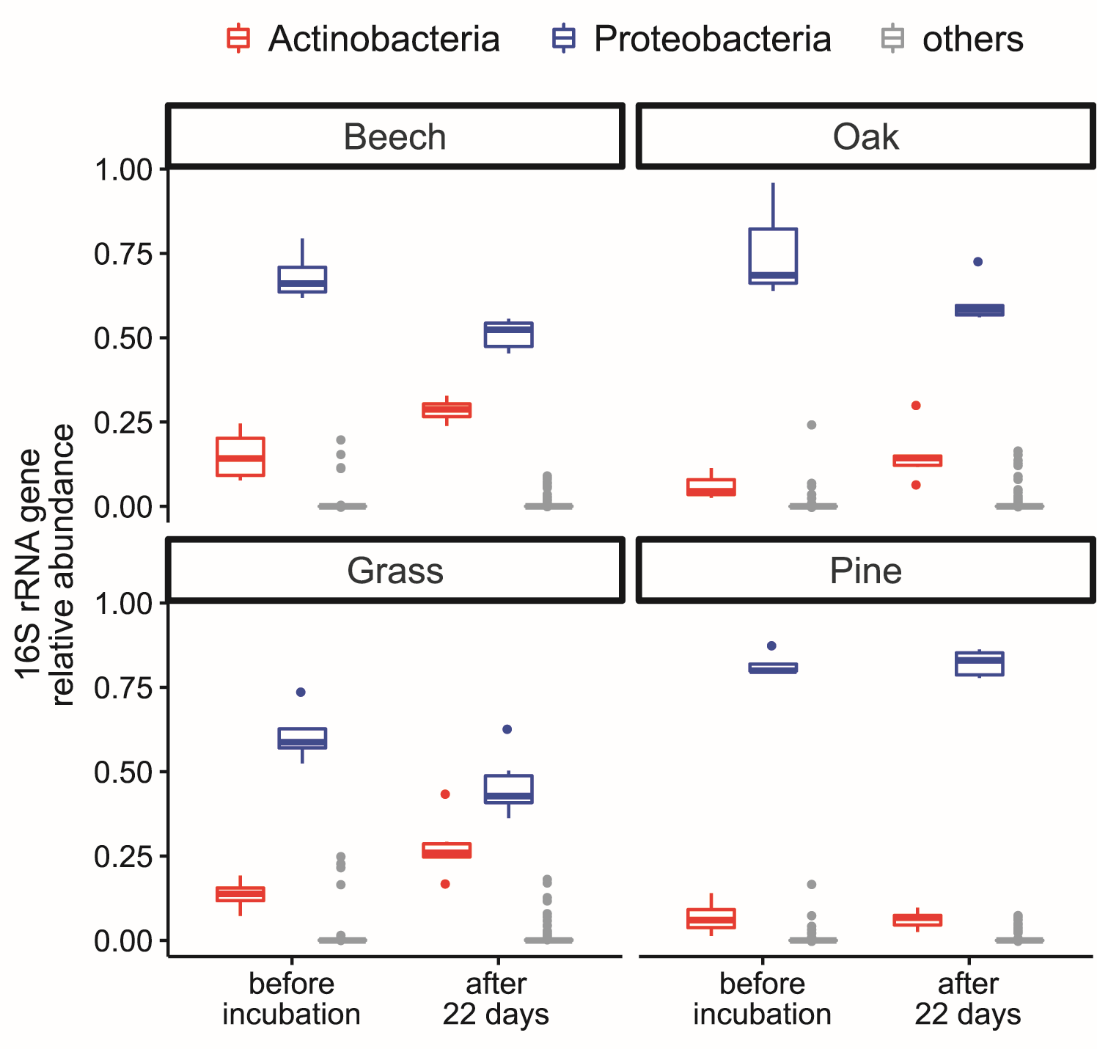


**Supplementary Figure S2** Relative proportions of *Proteobacteria* and *Actinobacteria* based on amplicon sequencing of the 16S rRNA gene.


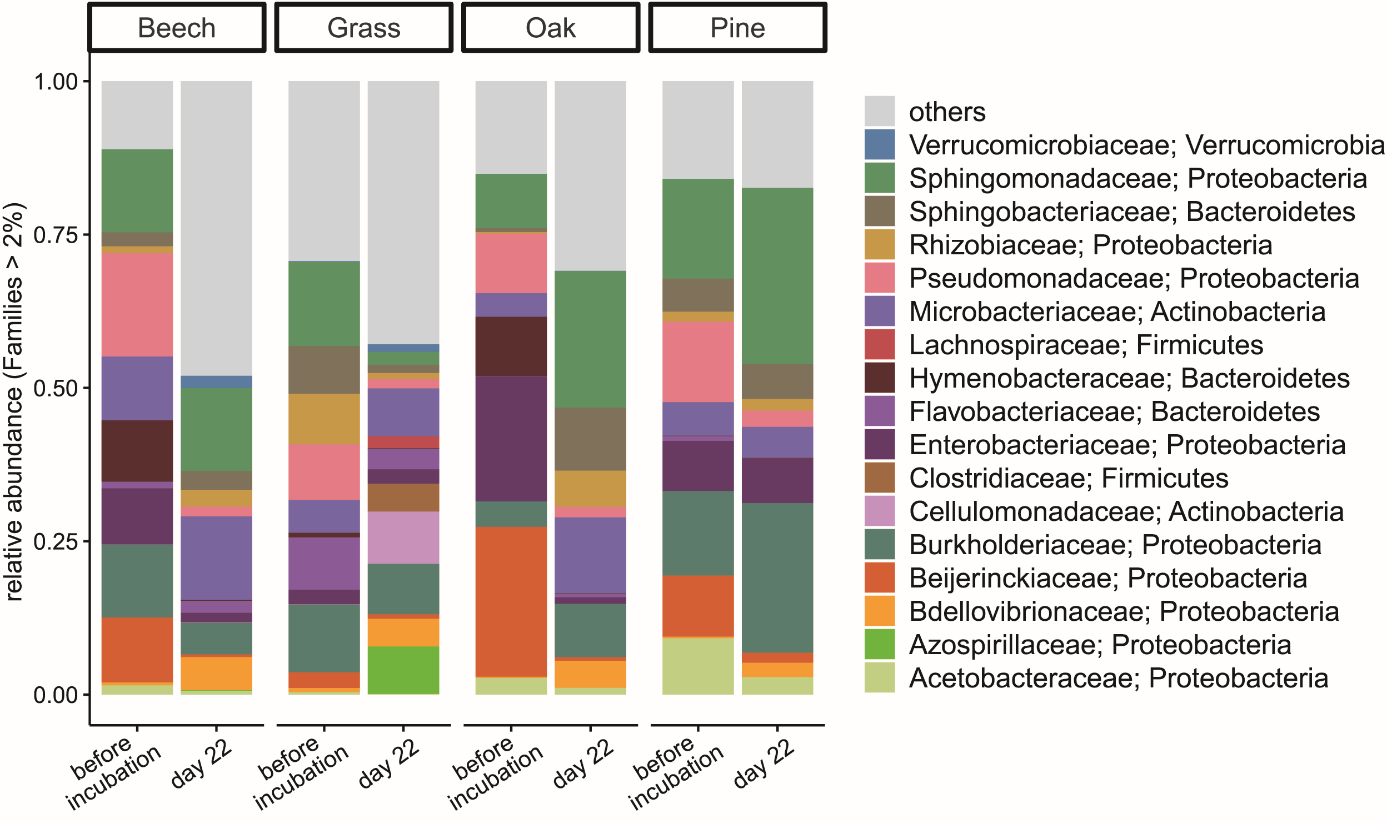
 **Supplementary Figure S3** Overview of the bacterial community composition based on amplicon sequencing of the 16S rRNA gene.
